# Expertise-Related Differences in Wrist Muscle Co-contraction in Drummers

**DOI:** 10.3389/fpsyg.2020.01360

**Published:** 2020-07-24

**Authors:** Scott Beveridge, Steffen A. Herff, Bryony Buck, Gerard Breaden Madden, Hans-Christian Jabusch

**Affiliations:** ^1^Institut für Musikermedizin (IMM), Hochschule für Musik Carl Maria von Weber, Dresden, Germany; ^2^Digital and Cognitive Musicology Lab (DCML), École Polytechnique Fédérale de Lausanne (EPFL), Lausanne, Switzerland; ^3^Music Cognition and Action Research Group (MCA), MARCS Institute for Brain, Behaviour & Development, Western Sydney University (WSU), Sydney, NSW, Australia

**Keywords:** electromyography, drummers, expertise, practice, motor control, coordination abilities, musicians, muscle co-contraction

## Abstract

**Background and Aim:** Drumming requires excellent motor control and temporal coordination. Deploying specific muscle activation patterns may help achieve these requirements. Muscle activation patterns that involve reciprocal contraction of antagonist muscles are particularly favorable as they enable a high level of muscular economy while maintaining performance. In contrast, simultaneous contraction of antagonist muscles is an inefficient muscle activation pattern. In drumming, co-contraction can lead to increased movement variability and greater fatigue over time. In this study we examine how muscle activation patterns develop with increased drumming expertise.

**Methods:** Eleven expert drummers (ED) and eleven amateur drummers (AD) were recorded using 3D motion capture while performing five different uni-manual and bi-manual repetitive drumming tasks across different tempi. Electromyography was used to record muscle activation of wrist flexor and extensor muscles.

**Results:** Findings indicate that reduced co-contraction resulted in more even drumming performance. Co-contraction also increased in extremely slow and very high tempi. Furthermore, regardless of task or tempo, muscle co-contraction was decreased in participants with higher levels of expertise. In addition to anti-phasic activity of wrist flexor and extensor muscles, expert drummers exhibited a flexor dominance, suggesting more efficient usage of rebound.

**Conclusion:** Taken together, we found that higher levels of drumming expertise go hand in hand with specific muscle activation patterns that can be linked to more precise and efficient drumming performance.

## 1. Introduction

Drumming requires highly coordinated, repetitive movements that are both accurate and energy efficient. Physiological energy efficiency is particularly important in order to satisfy the task demands associated with drumming performance (De La Rue et al., 2013). Traditional strategies for economic movement minimize metabolic energy expenditure by reducing joint stiffness. Movement around a joint is initiated by the agonist muscle or muscle group that acts as the “prime mover” in the motion. The opposing set of muscles, the antagonists, counteract this motion; co-activating both sets of muscles help to stabilize the joint (Freivalds, 2004; Bartlett, 2007). Joint stiffness results when agonist and antagonist muscles contract simultaneously (co-contract) and hence impede movement (Gribble et al., 2003). Professional musicians exhibit movement patterns that avoid muscle co-contraction and the associated joint stiffness (Furuya and Kinoshita, 2008; Fujii et al., 2009a; Verrel et al., 2013). A reduction in co-contraction during movement minimizes metabolic energy expenditure, reduces fatigue, and optimizes physical performance (Osu et al., 2002; Huysmans et al., 2008).

Whilst energy inefficient, co-contraction can have beneficial effects during skill acquisition. In the early learning stages of performing simple arm actions, such as pointing or reaching, the co-contraction of antagonistic muscle pairs is commonly observed and improves movement accuracy (Gribble et al., 2003; Wong et al., 2009). As skill increases, this co-contraction decreases while accuracy remains high (Bernstein, 1967; Moore and Marteniuk, 1986; Thoroughman and Shadmehr, 1999; Osu et al., 2002; Gribble et al., 2003). This reduction in muscle co-contraction has also been reported during the learning of music-related movements, including drumming (Fujii et al., 2009a,b; Verrel et al., 2013). Specifically, Fujii et al. (2009a,b) report pronounced reciprocal contractions of antagonistic muscle pairs acting on the wrist: the flexor carpi ulnaris (FCU) and the extensor carpi radialis (ECR). In addition, Fujii observed a shorter decline in muscle activity and a smaller variability of activation time of the wrist flexor muscle (flexor carpi ulnaris) in drummers when compared to non-drummers. Here, we aim to shed further light on expertise-related changes in muscle activation patterns during repetitive drumming using surface electromyography (sEMG; sEMG measures electrical activity on the surface of the skin that reflects activation of the underlying muscle groups).

Patterns of muscle activation can also be indicative of task difficulty. Chong and colleagues asked non-musicians to play hand percussion and showed that sEMG amplitude rise increased in response to increased playing tempo (Chong et al., 2015). Similar results were observed during keyboard playing, with sEMG activity rising in specific forearm muscles in response to increased tempo (Chong et al., 2015). Here, we also explore the role of tempo in muscle activity patterns, and how tempo-induced task difficulty may interact with expertise.

By measuring sEMG of 18 participants during a uni-manual rapid tapping task, Fujii and colleagues illustrated the relationship between expertise and muscle co-contraction. In line with previous literature, the authors observed levels of co-contraction decreasing as skill increased (Fujii et al., 2009b). In the present study, we also explore the connection between expertise and co-contraction. We aim to extend Fujii's findings to also include synchronous and alternating bi-modal movements, whilst controlling the drumming patterns across participants and conditions of tempo. Instead of a rapid tapping task (where participants are asked to tap as fast as possible), five 8-beat striking patterns are performed across standardized conditions of tempo. Controlling for striking patterns across participants allows a direct comparison of expertise-related muscle activity across tasks.

In order to measure drumming performance at different tempi, prior research required participants to play in synchrony with an external isochronous referent signal (e.g., a metronome). The temporal distance between consecutive taps, termed the inter-tap interval (ITI), is a useful metric for assessing tapping performance. If the participant taps at a constant tempo, the coefficient of variation of these inter-tap intervals (CV-ITI) will be low. Paced finger tapping research has shown significant differences in synchronization ability between musicians and non-musicians. This is particularly evident in the synchronization ability at very high and very low tempi. When compared to non-musicians, instrumentalists (including pianists, violinists, cellists, and drummers) can achieve faster ITIs with noticeably greater accuracy than their non-musically trained counterparts (Dahl, 2004, 2006; Krause et al., 2010; Repp, 2010b; Fujii et al., 2011). At lower tempi (metronome inter-onset intervals (IOIs) ranging from 1,000 to 3500 ms), musicians' taps are also less variable than those of non-musicians (Repp and Doggett, 2007). It is worth noting that upper rate performance is restricted primarily by the biomechanical capabilities of the end-effector (for instance, the maximum frequency that the finger can move) (Fujii et al., 2011), whereas lower rates are dictated by perceptual and cognitive time-keeping mechanisms (Repp and Doggett, 2007). At low tempi, interval subdivision has been reported to reduce synchronization error. Bisection of long time intervals can be achieved covertly (imagining a beat) or overtly (adding extra movements between beats) with overt strategies providing most benefit to non-musicians' timing precision.

Drumming studies have revealed that, in addition to precise control of limb movements, control of the end-effector plays a major factor in accuracy as well as influencing timbral aspects of the performance (Dahl and Altenmüller, 2008; Fujisawa and Miura, 2010). Drum strokes can be considered discrete actions, but more often linked together as a continuous motion. This allows for preparatory actions in the stick rebound phase that improve stick control (Dahl, 2011). Stick control is an important determinant of co-contraction (Dahl and Altenmüller, 2008; Fujisawa and Miura, 2010), which, in turn, has been connected to varying levels performance accuracy. In a study examining playing strategy and performance experience between amateur and non-drummers, Fujisawa and colleagues found that less skilled drummers played with higher levels of muscular strain (co-contraction) (Fujisawa and Miura, 2010). Furthermore, Kawakami and colleagues demonstrated that the sound and energy of the performance during repeated striking was directly related to the acceleration control of the stick both before and after each hit (Kawakami et al., 2008). Similar variations have been found between striking impulse and tempo control strategies of pianists. The use of such strategies influenced not only the tempo but also the tone of the notes (Furuya and Kinoshita, 2007).

The aim of this study is to examine the relationships between drumming performance, expertise, and muscle activity patterns using 3D motion capture and sEMG. We investigate how these relationships may be influenced by tempo-induced task difficulty. Muscle activation is recorded in (flexor-extensor) wrist muscle pairs. Precision is quantified by means of timing accuracy, examined across conditions of task and tempo. Our study is designed to examine associations between these factors and not causality.

## 2. Materials and Methods

### 2.1. Participants

Twenty-two drummers participated in the study. A group of expert drummers (“*ED*,” *N* = 11, 11 male) comprised mainly of students from the Jazz, Rock, Pop drumming department at the Dresden University of Music Carl Maria von Weber. All (a) had a minimum of 10 yrs drumming experience and (b) were actively participating in giving/receiving lessons and playing in local bands/ensembles at the time of study. The amateur drummer group (“*AD*,” *N* = 11, 8 male) comprised actively performing amateur drummers from the Dresden area. Inclusion in the amateur group required that participants (a) had a minimum of 1 year playing experience and (b) were currently participating in an ensemble or band. Twenty-one participants were confirmed to be right-handed using the Edinburgh Handedness Inventory, one had a tendency for being ambidextrous (Oldfield, 1971). Groups matched with regards to age but differed with respect to Age of Commencement (AoC) and Cumulative Lifetime Practice (CLP) (Table 1). Participants received € 20 for taking part in the study.

**Table 1:** ^†^Alpha levels for significance were predetermined at 0.05 (significant, *), 0.01, and 0.001 (highly significant, ** and ***, respectively).

	**Expert drummers (ED) *N* = 11**	**Amateur drummers (AD) *N* = 11**	
	**Median (min/max)**	**Median (min/max)**	**Mann–Whitney *U p*-value (2-tailed)^†^**
Age (yrs)	24.0 (19.4/40.1)	27.7 (21.1/34.4)	0.077
AoC (yrs)	9 (4/14)	14 (9/22)	**
YoP (yrs)	16 (9.6/35.1)	12.7 (3.0/21.4)	n.s.
CLP (1,000 h)	8.8 (3/15)	2.4 (0.5/6.9)	***

*Descriptive overview of relevant demographic data of the ED and AD groups. AoC, age of commencement of musical practice (drums); YoP, total years of practice (drums); CLP, cumulative life-time practice (from year-by-year daily/weekly practice according to retrospective self-reports)*.

### 2.2. Subject Posture and Task

After providing informed consent, participants were seated in front of a 12-inch neoprene practice pad (ProMar® X-Pad Snare Practice Pad). This pad resembles a snare drum with respect to handling and dimensions. While the height of the playing surface was kept fixed for all participants, the position and height of the drum stool was adjustable and left under the control of each participant. All subjects used an identical pair of drumsticks (Wincent®5B, weighing 64 g each). Participants were instructed to hold one stick in each hand and to perform the task using the “matched grip” technique (cf. Moeller, 1956). All tasks were to be performed at a constant loudness level between 55 and 65 dB(A). In order to become acquainted with the prescribed loudness, subjects were invited to play their own choice of warm-up task. During this warm-up task, visual feedback of their actual loudness was provided via the display of a digital sound pressure level meter (PCE Instruments® PCE-318). For the subsequent actual recording of the task, the visual feedback was removed.

**Figure A1:**
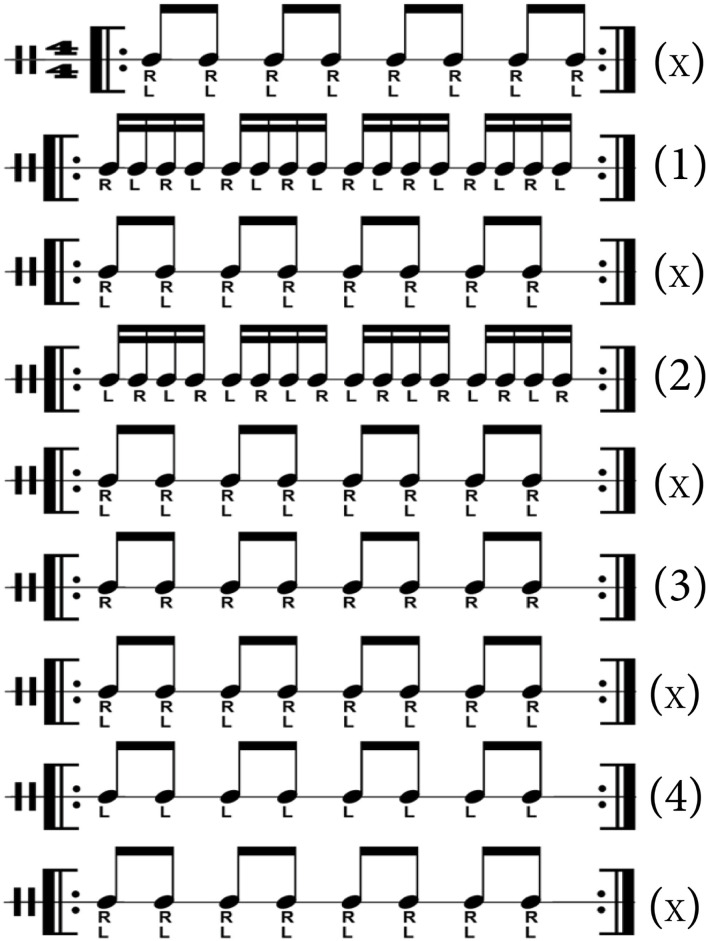
Drum patterns included in the 18-bar exercise (1) both hands alternating (BHRLead), with the right hand leading (i.e., right hand down stroke coinciding with the first beat of a measure), (2) both hands alternating (BHLLead), with the left hand leading (i.e., left hand down stroke coinciding with the first beat of a measure), (3) right hand solo (RHSolo), (4) left hand solo (LHSolo), (X) both hands simultaneously (BH), filler condition.

The experimental task involved playing an 18-bar drum exercise presented on a notated sheet in percussion notation (see Appendix A, Figure A1). The drum exercise comprised four 2-bar patterns of repetitive eighth notes in the following conditions: (1) both hands alternating (BHRLead), with the right hand leading (i.e., right hand down stroke coinciding with the first beat of a measure), (2) both hands alternating (BHLLead), with the left hand leading (i.e., left hand down stroke coinciding with the first beat of a measure), (3) right hand solo (RHSolo), (4) left hand solo (LHSolo). A filler condition (X), where both hands played simultaneously, was inserted at the beginning and end of each exercise, as well as in between each of the above mentioned conditions (1–4). This filler condition served to ease the transition points between conditions. The entire 18-bar sequence had to be played without interruption; in the event that a participant failed to manage the sequence in one take, an additional recording was made.

Each participant performed the task in five tempo settings guided by a metronome (Wittner Quartz Metronone, MT-50) with 40, 80, 120, 160, 200 beats per minute (BPM) for a quarter note. The respective desired eighth note repetition rates in each hand were 80, 160, 240, 320, 400 hits per minute (HPM), corresponding to inter-tap intervals (ITI) of 750, 375, 250, 187.5, 150 ms, respectively. Tempo conditions were presented sequentially, in the same order for each participant.

### 2.3. Data Acquisition

#### 2.3.1. Muscle Activity

Muscle activity was recorded using surface electromyography (sEMG). The sEMG activity was collected via a multichannel wireless sEMG system (Desktop DTS™, Noraxon Inc). Bipolar Ag/AgCl circular electrodes (10 mm diameter, inter-electrode distance = 20 mm) (Noraxon Dual Electrodes™) were attached at three positions on the lower arm; one on a wrist flexor [Flexor carpi ulnaris (FCU)], one on a finger flexor [Flexor digitorium superficialis (FDS)], and one on a wrist extensor [Extensor carpi radialis (ECR)]. The data collected from the Flexor digitorium superficialis (FDS) was not used in this study. Before the electrodes were attached, the skin was treated to reduce inter-electrode resistance. In this treatment, a small area of the skin was shaved, rubbed with abrasive paste, and cleaned with alcohol. sEMG signals were sampled at 1,500 Hz and synchronized with an external 30 Hz SMPTE clock.

#### 2.3.2. Drumstick Motion Trajectories

Motion trajectories of the drumsticks were recorded using a 3D motion capture system with six high-speed infrared cameras (Oqus 3, Qualisys, Sweden). Recordings were made at a frame rate of 500 fps and with a camera exposure time of 200 μs.

The locations for the placement of the passive infrared-reflective markers (12.5 mm diameter, Qualisys, Sweden) was similar to those in previous studies on drumming (Dahl, 2000, 2004, 2006; Waadeland, 2003, 2006). As this analysis does not include markers placed on the body, these will be reported elsewhere.

**Figure 1:**
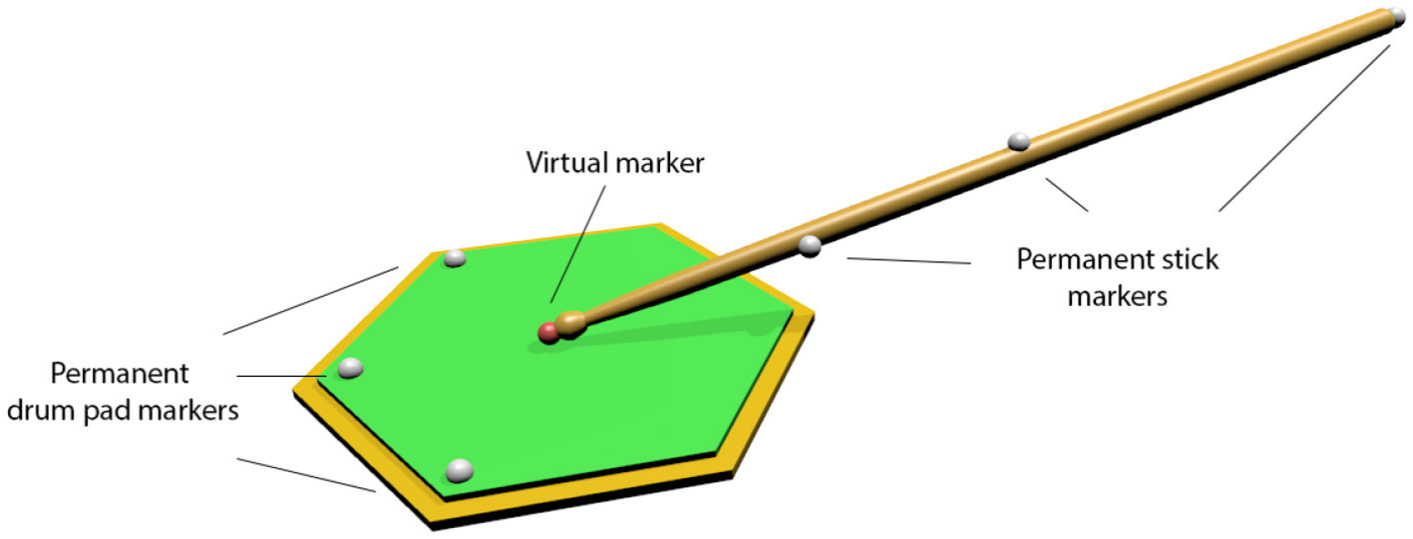
Schematic of the positions of the infrared-reflective markers on the drum stick and drum pad. Three permanent markers at the drumstick allowed modeling a “virtual marker” at the tip of the drumstick (red sphere). Three permanent markers on the surface of the drum pad allowed for precise 6DOF tracking of the interaction between stick tip and drum pad surface.

The drumsticks were permanently equipped with three markers (12.5 mm) each (Figure 1). These three markers were positioned in non-collinear fashion in order to allow for full six degree-of-freedom (6DOF) description of the stick motion including rotations of the stick around the long axis. Due to the mechanical forces acting on the tip of the stick upon impact during drumming, the stick tip could not be fitted with a permanent marker. We therefore used the 6DOF properties of the stick to create “virtual markers” at each stick tip. Virtual markers were created by performing static measurements with temporary 4 mm hemispheric markers at each stick tip which were subsequently removed. The recorded position of these temporary markers allows the exact spatiotemporal positions, velocities, and accelerations of the stick tip to be modeled. The playing surface (drum pad) was permanently equipped with three markers, again, allowing for precise 6DOF description of the interaction of the stick tip with the pad surface plane.

### 2.4. Data Processing and Analysis

Raw sEMG and 3D time series data from each subject was separated into individual epochs based on the five task conditions (BHRLead, BHLLead, RHSolo, LHSolo, BH). Each task condition comprised two four-beat measures. To omit movement transitions from (and into) the different tasks the first and last two strikes of each condition were removed. Each subject therefore provided 25 data sets (5 task conditions × 5 tempi, each set containing 12 drum strokes). Data sets comprised movement trajectory and corresponding (sEMG) muscle activity data. All data sets were included in the current analysis.

#### 2.4.1. Surface Electromyography

Raw sEMG signals were processed to remove zero-offset and band-pass filtered (low pass frequency/high pass frequency, 5/500 Hz). The resulting signals were then full-wave rectified and smoothed to calculate their corresponding envelopes. Smoothing was performed with a moving root-mean square (RMS) filter with a 9 ms window. This procedure was applied to both the extensor and flexor raw sEMG signals.

Raw sEMG signals were normalized to a reference value provided by the maximum voluntary contraction (MVC) of each muscle. The MVC provides maximum sEMG output, and, in accordance with Konrad (2005), constitutes a physiologically relevant calibration unit. To measure the MVC for each muscle (FCU, ECR), participants were instructed to perform an isometric maximum voluntary contraction against a static object. sEMG signals were measured in a “best of three” procedure, with a rest period of at least 30 s between contractions (De Luca, 1997; Halaki and Ginn, 2012). The resulting raw signals were post-processed in exactly the same manner as described above (zero-offset removal, band-pass filtering, etc). To find the peak value of the MVC signal (that represents the maximum voluntary contraction), a 500 ms sliding window was used to calculate the mean amplitude of the signal. This sliding window technique represents a more stable method as opposed to finding a single peak (Konrad, 2005; Halaki and Ginn, 2012).

##### 2.4.1.1. Relative difference signal

The Relative Difference Signal (RDS) was used to characterize co-contraction between flexor (FCU) and extensor muscles (ECR) (Heuer, 2007; Fujii et al., 2009b). This “compound measure” of muscle activity accounts for both phase and magnitude relationships between antagonistic muscle pairs. The RDS signal is calculated at each time point using the envelope signals of the extensor *e*(*n*) and flexor *f*(*n*) as follows:

(1) $$\begin{array}{l} RDS\left(n\right)=\frac{e\left(n\right)-f\left(n\right)}{e\left(n\right)+f\left(n\right)} \end{array}$$

where *e*(*n*) and *f*(*n*) are the time series corresponding to the envelopes of the extensor and flexor, respectively, and *n* is the time index. The RDS provides a distribution of the relative strength of each muscle over consecutive time points. According to Fujii et al. (2009b), we summarized the amount of co-contraction using the standard deviation of the RDS time series distribution (*sdRDS*).

**Figure 2:**
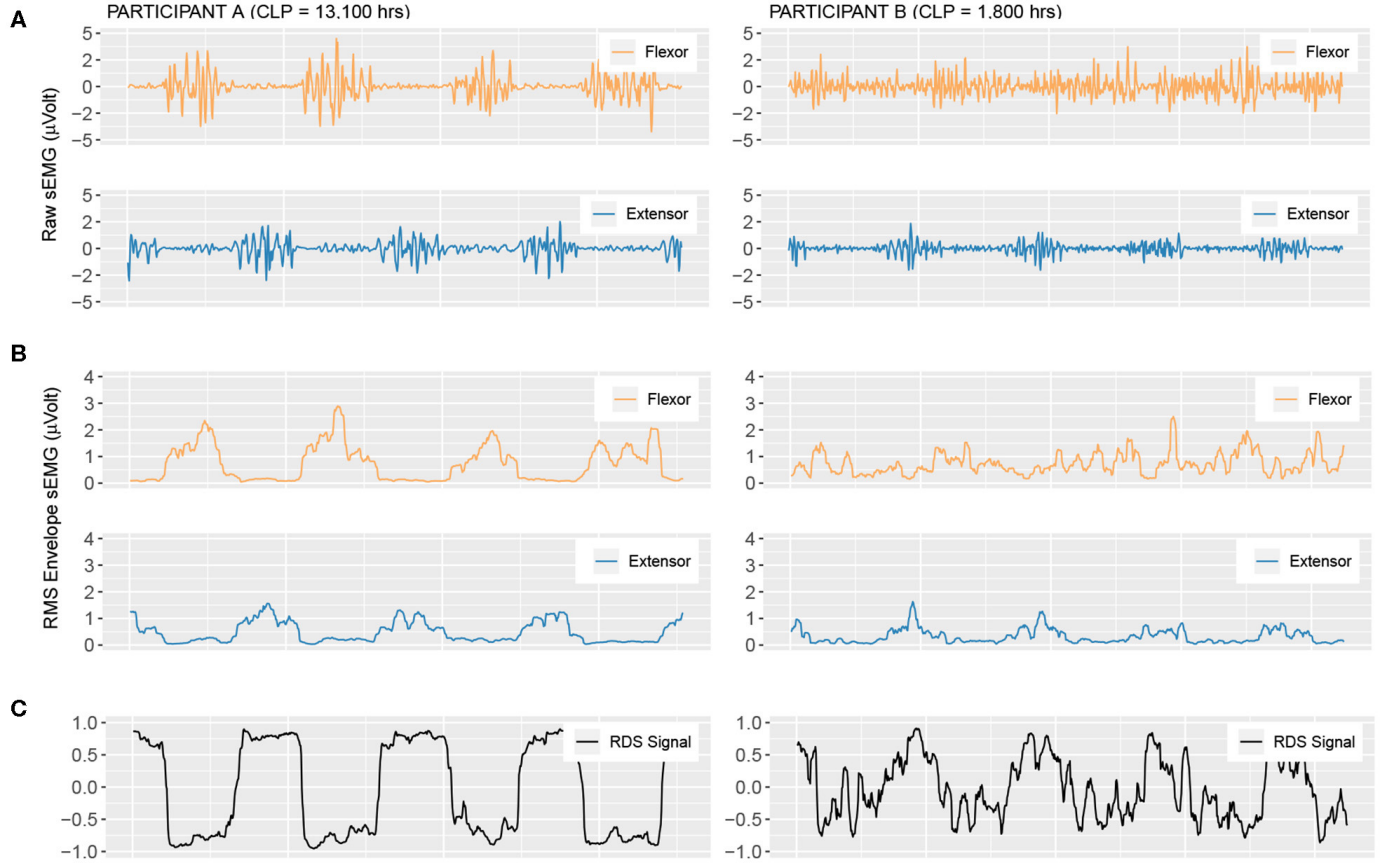
Surface electromyography signals for the single right-hand solo exercise (RHSolo) at 400 HPM. Participant A (left-hand column) is a member of the Expert group (ED) with a Cumulative Lifetime Practice (CLP) of 13,100 h. Participant B (right-hand column) is a member of the Amateur group (AD), with a Cumulative Lifetime Practice (CLP) of 1,800 h. **(A)** The raw sEMG signals of both flexor and extensor **(B)** The corresponding RMS Envelope signals. **(C)** The resultant relative difference signal (RDS).

**Figure 3:**
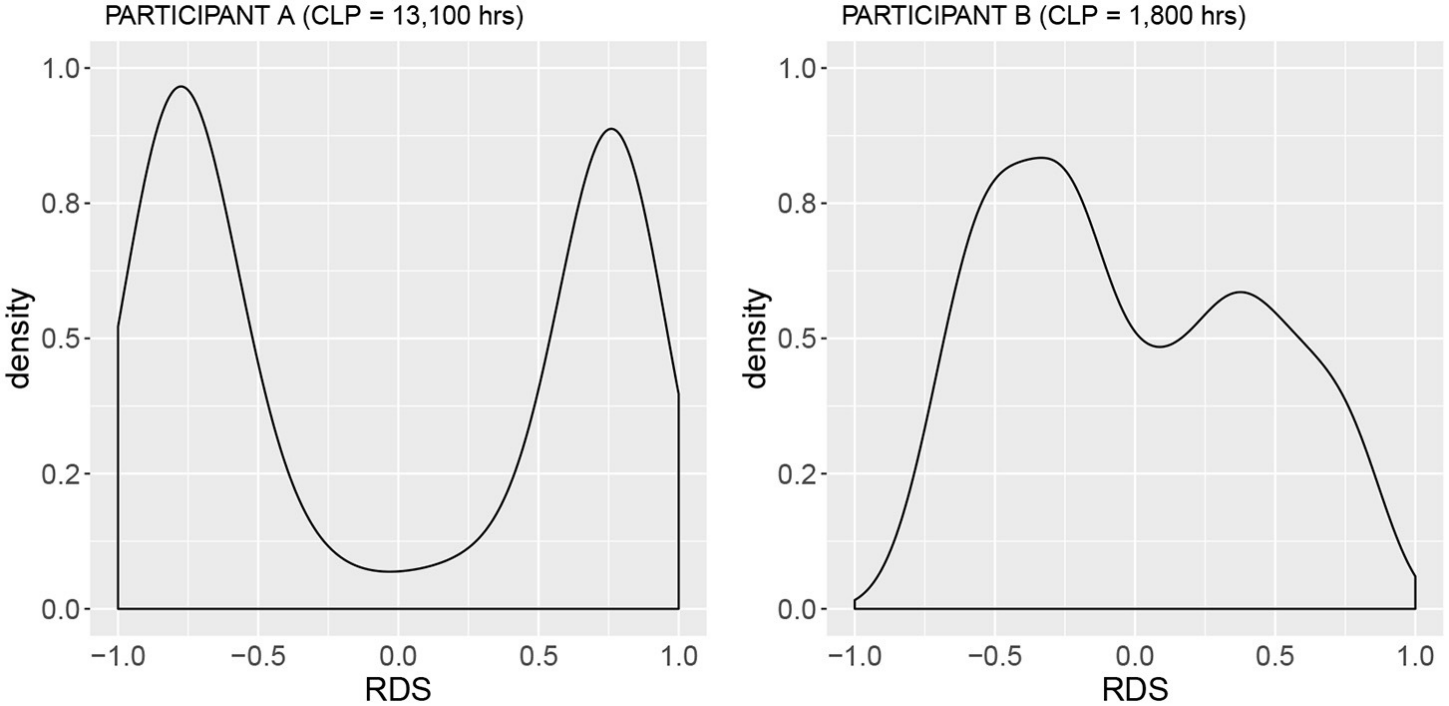
Density plots of the RDS signal for Participant A, Cumulative Lifetime Practice, CLP = 13,100 h, *sdRDS* = 0.74 and Participant B, Cumulative Lifetime Practice, CLP = 1,800 h) *sdRDS* =0.46. RDS signals from both plots were derived from the single right-hand only exercise (RHSolo) at 400 HPM (see Figure 2). Note the bi-modal distribution of Participant A, that accounts for the large standard deviation (*sdRDS*).

In Figure 2, we see the derivation of the relative difference signal from raw unprocessed sEMG (Figure 2A), the processed envelope signal (Figure 2B), and finally the resultant RDS signal (Figure 2C). Figure 3 shows the distribution of the RDS signal. Here, when co-contraction is low, the distribution becomes bi-modal with a tendency to assume values around −1 and 1. This results in a large standard deviation of the signal (high *sdRDS*). When the level of co-contraction is high, the distribution of the RDS signal will be unimodal and cluster around zero. This leads to a smaller standard deviation of the RDS signal (low *sdRDS*).

##### 2.4.1.2. Cross correlation coefficient

The RDS signal provides a compound measure of muscle co-contraction that examines sample-by-sample differences between flexor and extensor muscles comparisons over time. However, this measure does not allow phase and magnitude to be examined individually. In order to consider phase and magnitude separately, we use the cross-correlation method proposed by Fujii et al. (2009a).

As in Fujii et al. (2009a), the cross-correlation coefficient is calculated by shifting the envelope of the extensor *e*(*n*) with respect to the flexor envelope *f*(*n*). The amount of offset between the envelopes, or lag, is limited to the range between 0 ms and the mean inter-tap interval (ITI) for each exercise. The inter-tap interval is a time series defined as the differences between successive taps in each exercise (Further explanation in section 2.4.2). The following equation is used to calculate the cross-correlation coefficient between the envelopes of the flexor and extensor (Chatfield, 1984; Li and Caldwell, 1999):

(2) $$\begin{array}{l} r_{ef}\left(l\right)=\frac{\sum_{n=0}^{N}\left(e\left(n+l\right)-\mu_{e}\right)\left(f\left(n\right)-\mu_{f}\right)}{\sigma_{e}\sigma_{f}} \end{array}$$

where *l* is the lag, μ_*e*_ and μ_*f*_ are the means of *e*(*n*) and *f*(*n*), respectively, and σ_*e*_ and σ_*f*_ are their standard deviations.

##### 2.4.1.3. Phase

**Figure 4:**
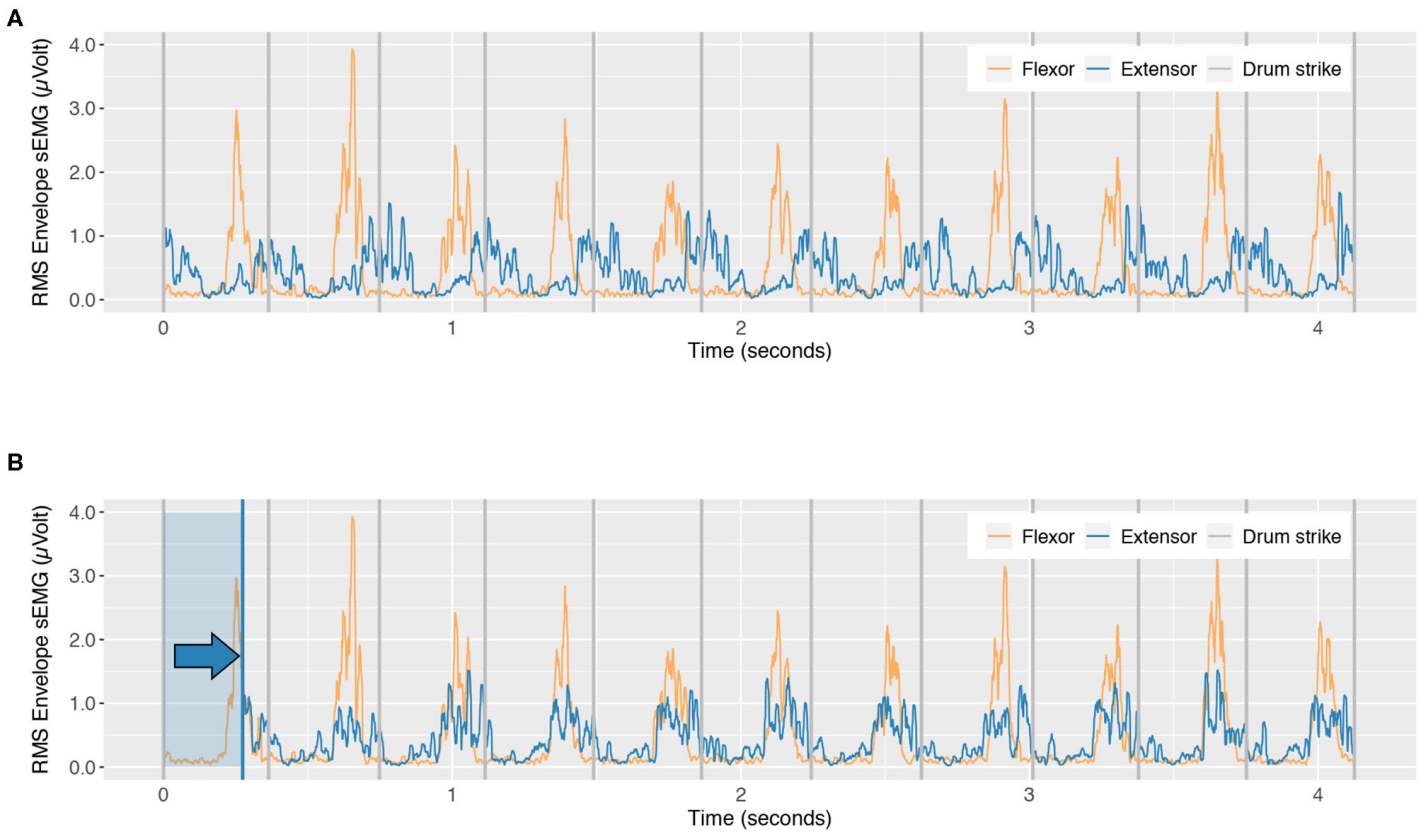
Derivation of phase shift from flexor/extensor cross correlation procedure. **(A)** Original signal envelopes. **(B)** Time shifted extensor signal showing the phase at peak cross-correlation of extensor and flexor (Blue shaded box and arrow shows the amount of phase shift).

To obtain the phase information, we calculate the maximum correlation coefficient and the corresponding time lag at this point (Figure 4). This gives a measure of the phase difference between peaks of muscle activity. We express this as a percentage of the mean inter-tap interval (ITI), where 0% indicates a 0° in-phase relationship between flexor and extensor, and 0.5 indicates a flexor and extensor that is 180° out of phase. Further analyses on phase were performed using the variable “distance to-antiphase” which will be defined in 3.1.1.

##### 2.4.1.4. Magnitude

To obtain the magnitude information, we calculated the sum of the point-wise flexor minus extensor muscle potentials at the lag identified by the maximum cross-correlation coefficient.

(3) $$\begin{array}{l} Magnitude=\overset{n}{\underset{n=1}{\sum }}f\left(n\right)-e\left(n\right) \end{array}$$

where *e*(*n*) and *f*(*n*) are the time series corresponding to the envelopes of the extensor and flexor, respectively, and *n* is the time index.

#### 2.4.2. Drumstick Motion Trajectories

To assess performance precision we measure timing variability of drum strikes. Drum strikes are extracted from the marker positions of stick tip and drum pad surface plane described in section 2.3.2. The elapsed time between drum strikes (or drum tap) is defined as the inter-tap interval (ITI). The coefficient of variation of the inter-tap intervals (ITI) for each individual participant was obtained from the set of 12 strikes during each condition (BHRLead, BHLLead, RHSolo, LHSolo, BH). The coefficient of variation of the inter-tap intervals (CV-ITI) is calculated by dividing the standard deviation of the ITIs by the mean of the ITI across the set. This yields a relative value of ITI variation (expressed as a fraction of a musical eighth note, in percent, rather than absolute milliseconds). The use of relative deviations renders the measure independent of the prescribed tempo (which varies as a task parameter), allowing for statistical comparisons across tempi. Additionally, it provides a measure independent from the actual played, rather than prescribed, tempo since it is expressed relative to the mean ITI. Small values of CV-ITI indicate high rhythmic evenness and vice versa.

## 3. Results

### 3.1. Muscle Activity

We deployed generalized linear mixed effects models predicting *Expertise* [Expert drummer (ED) vs. Amateur drummer (AD)] based on *sdRDS*, as well as the sdRDS*Tempo (80, 160, 240, 320, 400 HPM) interaction. The 240 HPM (120 BPM) condition served as baseline. The model was also provided with random effects for *Exercise, Hand*, and *Muscle*. In general, all models deployed here attempted to achieve the maximal random effect structure as justified by the design, while avoiding singular fit (Barr et al., 2013).

**Figure 5:**
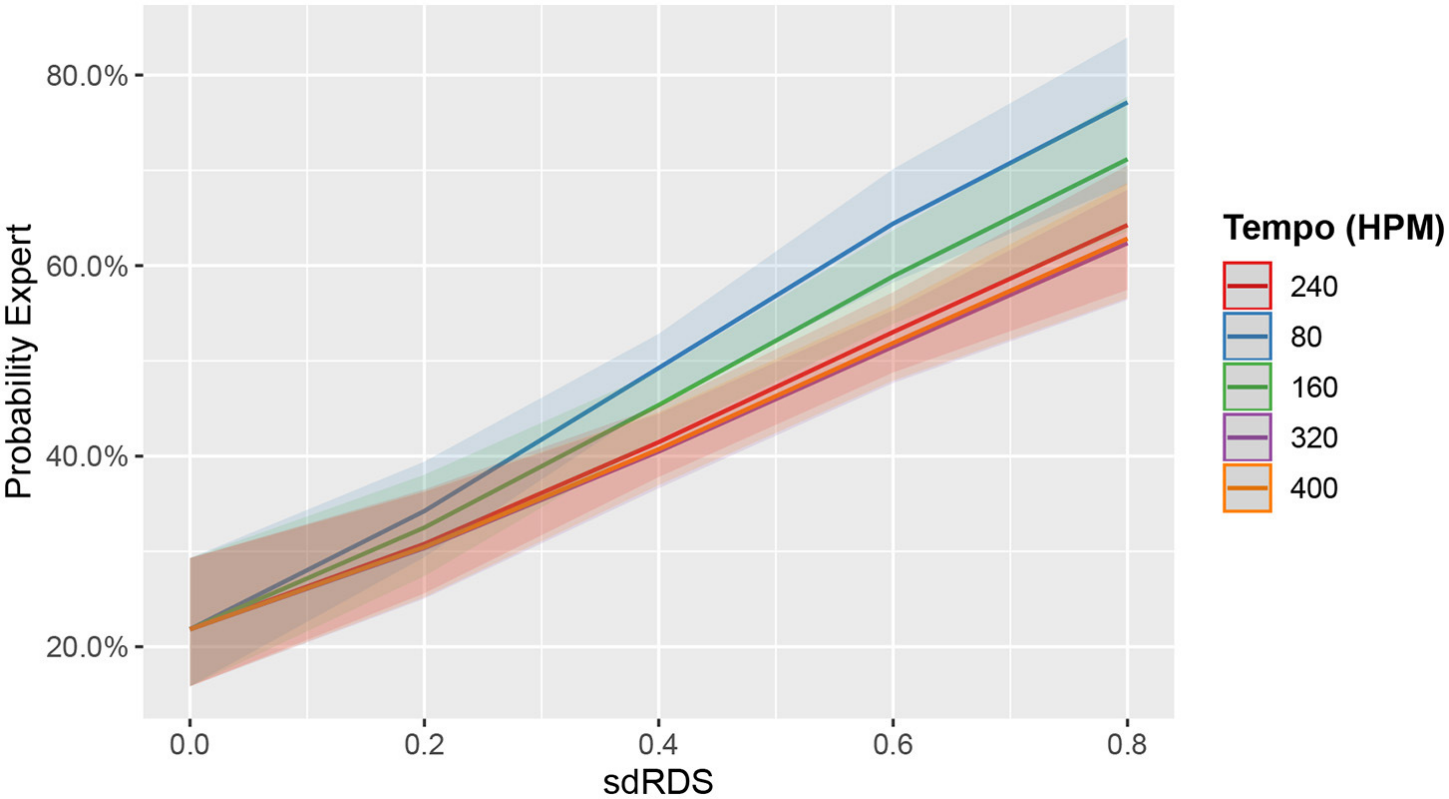
Marginal effects plot of *sdRDS* and tempo on the predicted probability that an observation derives from an expert drummer. Line color indicates tempo. The 240 HPM tempo is at the top of the legend, as it represents the model's baseline. The model attributes higher *sdRDS* to drumming expertise. This is particularly visible at 80 HPM (blue line), which shows the steepest slope. Bands represent 95% CIs.

Higher *sdRDS* significantly increased the probability that an observation derives from an expert (ED) rather than an amateur drummer (AD) (*Est*. = 2.32735, *SE* = 0.39365, *p* <.0001). This effect is exacerbated at 80HPM (*Est*. = 0.78669, *SE* = 0.24808, *p* = 0.00152). In 160, 320, and 400 HPM, the effect of *sdRDS* is comparable to 240 HPM (all *p* > 0.0642). Figure 5 depicts the marginal effects of *sdRDS* on model predictions for each tempo condition.

#### 3.1.1. Phase and Magnitude

**Figure 6:**
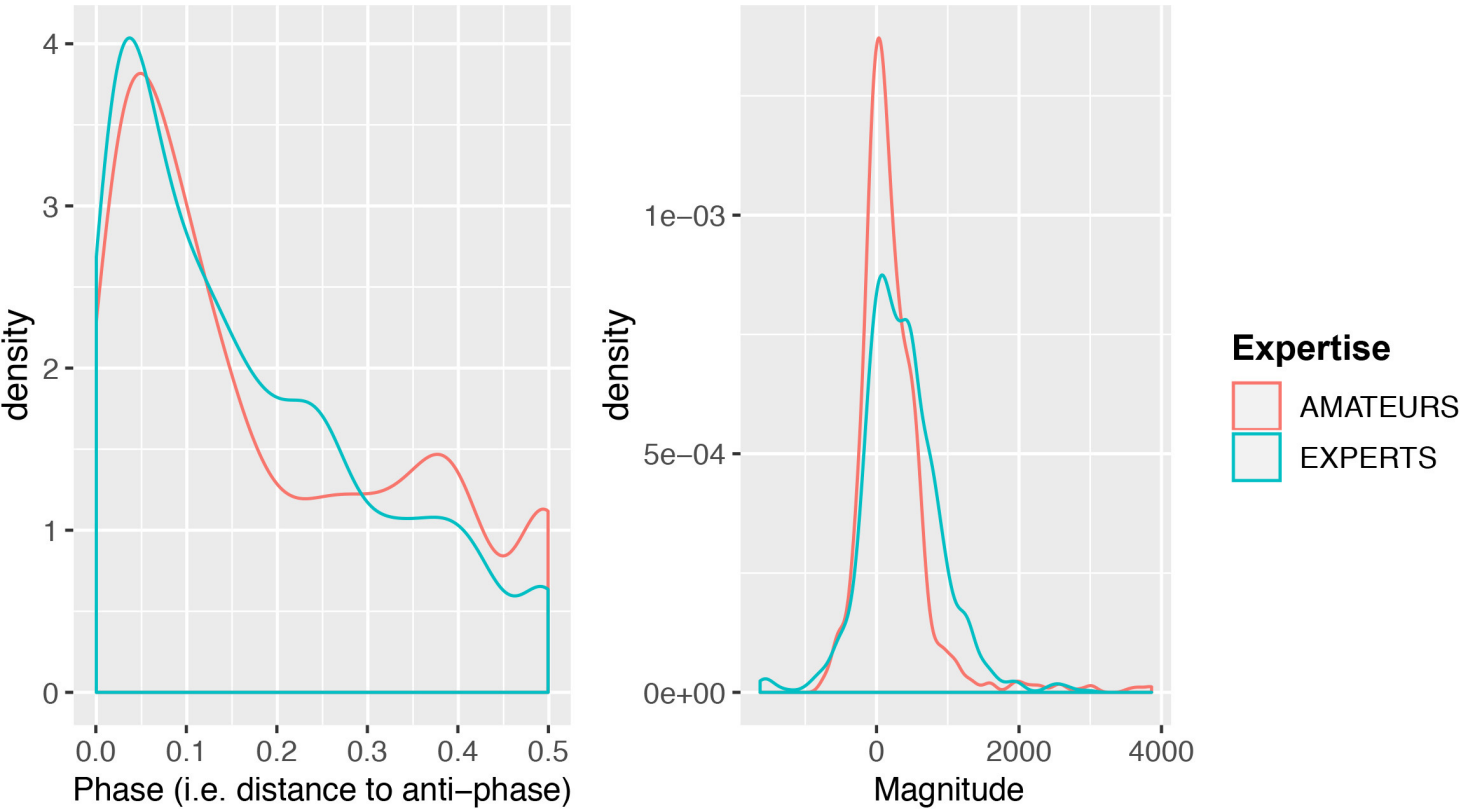
Density plot of relative phase (coded as distance to anti-phase) and magnitude in flexor and extensor muscles between the two expertise groups.

*sdRDS* is a composite measurement that draws from phase and magnitude information. As a result either or both could be driving the significant relationship between *sdRDS* and *Expertise* observed in the first model. Figure 6 is a density plot of the phase and magnitude distributions in experts and amateurs and provides a first insight into expertise-related differences in relative muscle activity. In the density plot, phase information is captured by the peak of the cross-correlation between flexor and extensor, coded as its relative distance to anti-phase. As a result a value of 0 represents anti-phase, and a value of 0.5 indicates in-phase muscle activity. The plot reveals a stronger tendency in expert drummers to play at anti-phase compared to amateur drummers. This can be seen by the higher blue line-height in the phase plot compared to the red line around 0. Simultaneously, the plot shows a stronger tendency to play in-phase in the amateurs. This can be seen by the higher red line-height in the phase density plot around 0.5 phase. For all further analyses, the variable *Phase* will be coded as “distance to anti-phase.”

The magnitude value is the sum of the point-wise flexor minus extensor muscle potentials at the lag identified by the maximum cross-correlation coefficient. In terms of relative magnitude, the density plot provides preliminary evidence that experts engage the flexor muscles more than the extensor muscles. This can be seen in the right-shifted magnitude distribution of the experts. The amateurs, in contrast, show a strong tendency to engage flexor and extensor muscles to the same extent, as indicated by the stark peak around 0 in the red line of the magnitude density plot.

In order to statistically disentangle the contribution of phase and magnitude we built an additional mixed effects model, this time predicting *Expertise* based on *Phase* and *Magnitude*, as well as their interaction. Again, the model was also provided with a random effect for *Exercise, Hand*, and *Muscle*. Phase information was coded as its absolute distance to anti-phase. Both fixed effects were standardized to *M* = 0, *SD* = 1.

**Figure 7:**
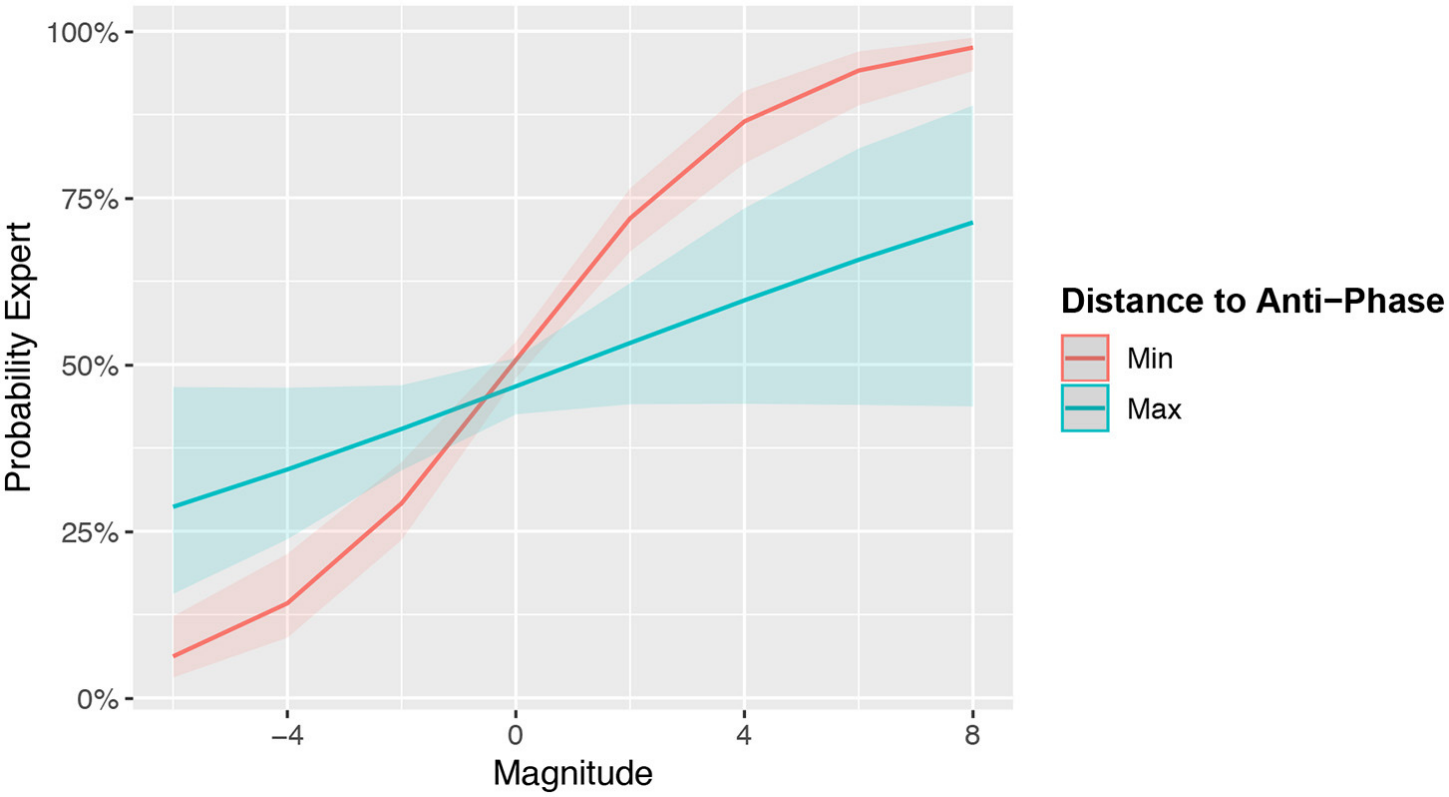
Marginal effects plot the Magnitude and Distance to Anti-Phase interaction on the predicted probability that an observation belongs to an expert. The model attributes muscle activity toward anti-phase and stronger flexor compared to extensor activity to expert drummers. Bands represent 95% CIs.

The model showed that *Magnitude* (*Est*. = 0.33915, *SE* = 0.04, *p* < 0.0001) predicted *Expertise*. *Phase* on its own did not predict *Expertise* (*Est*. = −0.04751, *SE* = 0.03672, *p* = 0.2). However, this is likely because the predictive information of *Phase* was captured in the significant Magnitude*Phase interaction term (*Est*. = −0.09809, *SE* = 0.03088, *p* = 0.0015). Specifically, the higher the relative activation of the flexor outweighs the extensor muscles, the more likely an observation was produced by an expert. However, this prediction becomes increasingly diminished as the muscles operate away from anti-phase. This can be seen in Figure 7.

### 3.2. Performance

In the last step, we investigated the actual drumming performance as measured by *CV-ITI*. Specifically, we deployed a linear mixed effects model predicting *CV-ITI* based on *CLP* (cumulative lifetime practice in hours), *sdRDS, Phase, Magnitude*, as well as *Tempo*. Random effects for *Exercise* and *Hand* were added to control for additional variability in the data. Since some of these factors contain overlapping information, this model allows us to explore whether *sdRDS* or the two separate *Magnitude* and *Phase* predictors contain more predictive information for performance. Note that here we are not concerned with interaction effects amongst those predictors, which is beyond the scope of this study. Again, the models base line was at the 240 HPM condition. Conservative *p*-values were obtained through Kenward-Roger approximations (Kenward and Roger, 1997).

**Figure 8:**
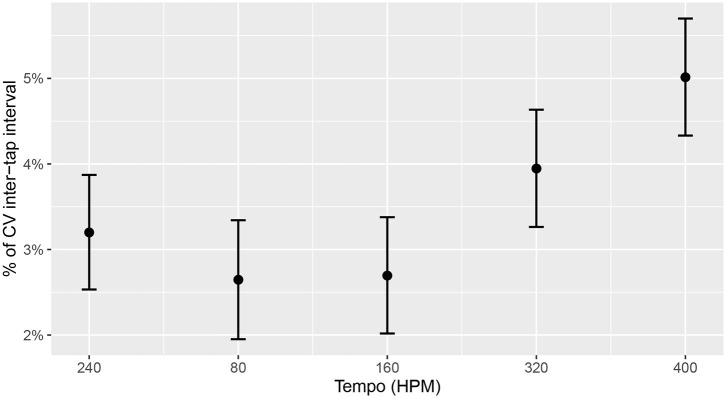
Predicted change in CV-ITI based on Tempo. Higher tempi show significantly less stable performances. Error bars represent 95% CIs.

The model predicted significantly higher *CV-ITI* at 320 HPM (*Est*. = 7.465413e-3, *SE* = 2.969159e-3, *p* = 0.01279233) and 400 HPM (*Est. = 1.813213e-2, SE = 2.992835e-3, p* < *0.0001)* compared to the 240 HPM base-line. This indicates that the drummers struggled more with playing evenly at high tempi compared to low tempi, regardless of task. This can also be seen in Figure 8.

**Figure 9:**
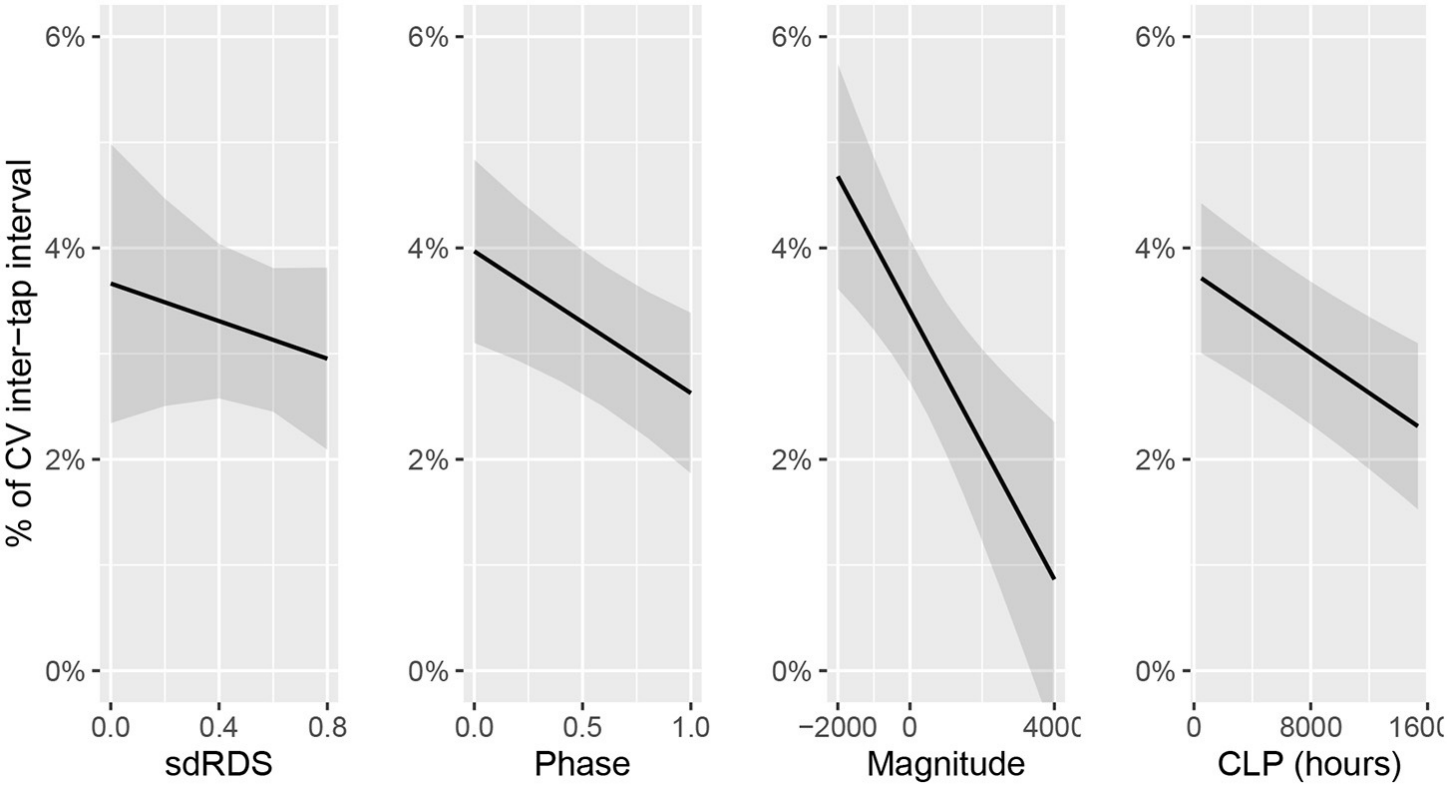
Predicted change in CV-ITI based on sdRDS, Phase, Magnitude, and CLP. The plot shows that more CLP predict lower CV-ITI. Stronger relative flexor activity compared to extensor activity, as well as engagement of flexor and extensor muscles in anti-phase also predict steadier performance. The predictive power of sdRDS seems to be better captured in Phase and Magnitude separately. Bands represent 95% CIs.

*CLP* significantly (*Est*. = −9.437871e-7, *SE* = 2.226901e-7, *p* < 0.0001) reduced *CV-ITI*. This suggests that increased training leads to increased temporal consistency in drumming. In Figure 9, this is depicted by the steep downward slope in CLP.

In terms of the link between electromyography and drumming performance, we observed that *Phase* and *Magnitude* both predicted lower *CV-ITI*. This indicates that the more the muscles operate toward anti-phase, and the stronger flexor activity outweighs extensor activity, the steadier the drumming performance. Furthermore, this finding suggests that whilst the composite measure *sdRDS* is a useful overall marker, *Phase* and *Magnitude* separately contain valuable information. This can be seen in Figure 9 by the steep downward slope in phase and magnitude and shallow line in *sdRDS*.

To further examine additional predictive information in *Phase* and *Magnitude* over *sdRDS* we used a model comparison approach. Specifically, we compared two models similar to the one described above. One of these models was provided with *sdRDS* but not *Phase* and *Magnitude*, whereas the other model was provided with *Phase* and *Magnitude* but not *sdRDS*. As can be seen in Table 2, the *Phase* and *Magnitude* model significantly (*X*^2^ = 15.797, *p* < 0.0001, ΔBIC = 8.4) outperforms the *sdRDS* model.

**Table 2:** Model comparison between sdRDS and Phase+Magnitude.

**Predictors**	**DF**	**AIC**	**BIC**	**LogLik**
*sdRDS*	10	−6457.0	−6402.2	3238.5
*Phase*+*Magnitude*	11	−6470.8	−6410.6	3246.4

*The Phase+Magnitude model outperforms the sdRDS model*.

## 4. Discussion

In this study we investigated the relationship between muscle co-contraction, expertise, and performance in a range of drumming exercises. The exercises included both uni- and bi- manual tasks and were performed at a range of tempi. Examination of flexor/extensor muscle phase and magnitude relationships uncovered patterns of reduced co-contraction linked to increased expertise. Specifically, there was an increased tendency for reciprocal contraction of muscles in those with high expertise. In previous studies this has been observed during rapid uni-manual tapping in groups of drummers and non-drummers (Fujii et al., 2009a,b). This work extends this finding, and confirms that it is generalizable across a continuum of expertise, over a range of tempi, and during both uni- and bi-manual drum exercises.

### 4.1. Muscle Co-contraction

Co-contraction during drumming was highly predictive of expertise level. This was evident in both the compound measure derived from the standard deviation of the relative difference signal (*sdRDS*), and by phase and magnitude separately. In general, drummers with high levels of expertise exhibited lower levels of muscle co-contraction. This was true across all exercises, hands (dominant/non-dominant), and tempi, but was especially prevalent at the lowest tempo in the study (80 HPM, ITI = 750 ms).

#### 4.1.1. Rebound Control

Analysis of the relative magnitudes of flexor and extensor muscle activity revealed a flexor dominance in high expertise drummers, compared to amateur drummers. This flexor dominance is highly indicative of expertise during anti-phase muscle activity, but diminishes the further the muscle activity moves from anti-phase. This flexor dominance can be explained by a more efficient use of rebound in high expertise drummers. The flexor carpi ulnaris, known as the “prime mover” in wrist flexion, is responsible for the initiation of movement that sets the stick in motion toward the drum pad. The extensor helps to stabilize the wrist and end effector (in this case the drum stick) on the upwards rebound trajectory. Our findings indicate that expert drummers expend less muscular energy on the rebound than amateur drummers. This motion pattern is less energetically expensive, more efficient and may lead to improved performance and accuracy.

A possible contributing factor of increased co-contraction at low tempo may be difficulties in stick rebound control. At high tempi, stick rebound can be incorporated into the preparatory action for the following drum stroke (Dahl, 2011). This groups consecutive strokes as a continuous stream of motion. However, at slower tempi the strokes are too far apart to use rebound in preparation for the next strike. Effectively this results in drum strokes that are discrete events. In these discrete movements, the stick must be brought to a stop, held in mid air, and then lifted to the starting position before the beginning of the next strike. These additional movements increase muscle activity and may account for increased co-contraction observed within both groups at the lowest tempo. Previous studies have shown that co-contraction increases under destabilizing conditions (Thoroughman and Shadmehr, 1999; Milner, 2002; Miura et al., 2013). It is possible that during these discrete strokes, drummers are also required to control non-muscular forces (inertial or gravitational forces) leading to the additional muscle activity and co-contraction observed in the present study. This type of additional activity has been previously observed during reaching actions, and is known as “wasted contraction” (Thoroughman and Shadmehr, 1999). In traditional drumming pedagogy, rebound control is often highlighted as being important in order to maximize energy efficiency and improve movement control (Logozzo, 1993; Famularo and Bergamin, 1999; Mayer, 2007). The present findings are consistent with previous research that found a pronounced flexor dominance in highly expert drummers (Fujii and Moritani, 2012), especially under discrete, single stroke drumming conditions (Fujisawa and Miura, 2010). Our findings also support the observation of increased co-contraction amongst those with lower level of drumming expertise (Fujii et al., 2009a,b).

#### 4.1.2. Time Keeping Movements

At the lowest playing tempo, we observed a tendency for performers to execute additional time-keeping movements between drum strikes. This tendency to subdivide large time intervals has been shown to reduce timing variability in tapping studies (Repp, 2010a) and may be a result of reported difficulties in tapping at slow tempi (Bååth and Madison, 2012). The implication for this study is that overt additional movements, manifesting as extra “mid-air” drum strikes reduce timing variability. However, they also result in additional muscle activity and potentially increased co-contraction.

### 4.2. Performance Accuracy

#### 4.2.1. Performance and Muscle Activity

Anti-phase muscle activity as well as flexor dominance predicted better drumming performance, as manifested in lower CV-ITI. A likely interpretation of these results presents itself in the literature on motor skill acquisition in drummers (Fujii et al., 2009a,b).

When expertise is low, co-contraction is deployed to stabilize performance (Bernstein, 1967). As expertise increases, so does performance quality, and alternative muscle activity patterns can be adopted that avoid co-contraction in favor of energy efficiency. In the present case, highly expert drummers tend to exhibit anti-phase muscle activity patterns. Although not always explicitly stated in these terms, the benefits of flexor dominance and reciprocity, and their relation to anti-phasic muscle activity, are extremely important in modern drumming pedagogy (Logozzo, 1993; Famularo and Bergamin, 1999; Mayer, 2007).

#### 4.2.2. Performance and Tempo

Our findings are consistent with previous studies that report performance of expert drummers (measured in CV-ITI) to range between 2 and 5% of an eighth note (Madison, 2000). However, across all drummers, expertise, and exercises we observed a significant drop in performance as tempo increased. A possible explanation for this is that we are reaching the biomechanical limits for rapid upper arm movement. The maximum tapping frequency of motor effectors are reported to be between 5 and 7 Hz, corresponding to ITIs of 150–200 ms. The highest tempo in the present study is 400 HPM, which represents an ITI of 150 ms. This may have prevented players (especially those with lower expertise) from fulfilling the task demands resulting in reduced performance accuracy (Fujii et al., 2011).

## 5. Conclusion

This study investigated the relationship between expertise, muscle activation, and performance in drummers. Our findings show that as drummers gain expertise, they tend to exhibit a muscle activation pattern that involves reciprocal firing of antagonist flexor/extensor muscles. This results in an overall reduction in muscle co-contraction and increase in drumming performance. These activation patterns are evident across a wide range of tasks, tempi, and during both uni- and bi-manual drumming exercises.

## Data Availability Statement

The datasets for this article are not publicly available because public availability of data is not covered by the ethics application and the ethics approval provided for this study [Institutional Review Board of the TU Dresden (IRB 00001473; EK 507122016)]. Requests to access the datasets should be directed to Scott Beveridge (beveridges@ihpc.a-star.edu.sg).

## Ethics Statement

The studies involving human participants were reviewed and approved by the Institutional Review Board of the TU Dresden (IRB 00001473; EK 507122016). The participants provided their written informed consent to participate in this study.

## Author Contributions

SB conducted the experiment, performed the analysis, and wrote and reviewed the manuscript. SH performed the statistical analysis and wrote and reviewed the manuscript. BB and GM reviewed the manuscript. H-CJ conceived and designed the study and wrote and reviewed the manuscript. All authors contributed to the article and approved the submitted version.

## Conflict of Interest

The authors declare that the research was conducted in the absence of any commercial or financial relationships that could be construed as a potential conflict of interest.
