# Corrigendum: Expertise-Related Differences in Wrist Muscle Co-contraction in Drummers

**DOI:** 10.3389/fpsyg.2021.706228

**Published:** 2021-07-23

**Authors:** Scott Beveridge, Steffen A. Herff, Bryony Buck, Gerard Breaden Madden, Hans-Christian Jabusch

**Affiliations:** ^1^Institut für Musikermedizin (IMM), Hochschule für Musik Carl Maria von Weber, Dresden, Germany; ^2^Digital and Cognitive Musicology Lab (DCML), École Polytechnique Fédérale de Lausanne (EPFL), Lausanne, Switzerland; ^3^Music Cognition and Action Research Group (MCA), MARCS Institute for Brain, Behaviour & Development, Western Sydney University (WSU), Sydney, NSW, Australia

**Keywords:** electromyography, drummers, expertise, practice, motor control, coordination abilities, musicians, muscle co-contraction

In the original article there were several errors in the text.

In the original article we stated that:

“In the early learning stages of performing simple arm actions, such as pointing or reaching, the co-contraction of antagonistic muscle pairs is commonly observed and improves movement accuracy (Fitts, 1954; Gribble et al., 2003; Yang et al., 2007; Wong et al., 2009).”

The references (Fitts, 1954) and (Yang et al., 2007) do not address specifically muscle co-contraction and have been removed.

A correction has been made to *Introduction, Paragraph 2*. The corrected paragraph is shown below.

Whilst energy inefficient, co-contraction can have beneficial effects during skill acquisition. In the early learning stages of performing simple arm actions, such as pointing or reaching, the co-contraction of antagonistic muscle pairs is commonly observed and improves movement accuracy (Gribble et al., 2003; Wong et al., 2009). As skill increases, this co-contraction decreases while accuracy remains high (Bernstein, 1967; Moore and Marteniuk, 1986; Thoroughman and Shadmehr, 1999; Osu et al., 2002; Gribble et al., 2003). This reduction in muscle co-contraction has also been reported during the learning of music-related movements, including drumming (Fujii et al., 2009a,b; Verrel et al., 2013). Specifically, Fujii et al. (2009a,b) report pronounced reciprocal contractions of antagonistic muscle pairs acting on the wrist: the flexor carpi ulnaris (FCU) and the extensor carpi radialis (ECR). In addition, Fujii observed a shorter decline in muscle activity and a smaller variability of activation time of the wrist flexor muscle (flexor carpi ulnaris) in drummers when compared to non-drummers. Here, we aim to shed further light on expertise-related changes in muscle activation patterns during repetitive drumming using surface electromyography (sEMG; sEMG measures electrical activity on the surface of the skin that reflects activation of the underlying muscle groups).

Next, in the original article we stated that:

“The pattern of motion employed and the characteristics of firing of muscles within the upper limbs was seen to increase the smoothness and accuracy of the striking motion executed (Gribble et al., 2003; Aluru et al., 2014)”.

The inclusion of this sentence is not within the context of the paragraph (musical movements) and has therefore been removed.

A correction has been made to *Introduction, Paragraph 3*. The corrected paragraph is shown below.

Patterns of muscle activation can also be indicative of task difficulty. Chong and colleagues asked non-musicians to play hand percussion and showed that sEMG amplitude rise increased in response to increased playing tempo (Chong et al., 2015). Similar results were observed during keyboard playing, with sEMG activity rising in specific forearm muscles in response to increased tempo (Chong et al., 2015). Here, we also explore the role of tempo in muscle activity patterns, and how tempo-induced task difficulty may interact with expertise.

Additionally, in the original article we stated that:

“Drumming studies have revealed that, in addition to precise control of limb movements, control of the end-effector plays a major factor in accuracy as well as influencing timbral aspects of the performance (Osu et al., 2002; Gribble et al., 2003; Dahl and Altenmüller, 2008; Fujisawa and Miura, 2010; Aluru et al., 2014).”

The inclusion of the references (Osu et al., 2002), (Gribble et al., 2003), and (Aluru et al., 2014) fall outside the scope of this paragraph and have been removed.

A correction has been made to *Introduction, Paragraph 6*. The corrected paragraph is shown below.

Drumming studies have revealed that, in addition to precise control of limb movements, control of the end-effector plays a major factor in accuracy as well as influencing timbral aspects of the performance (Dahl and Altenmüller, 2008; Fujisawa and Miura, 2010). Drum strokes can be considered discrete actions, but more often linked together as a continuous motion. This allows for preparatory actions in the stick rebound phase that improve stick control (Dahl, 2011). Stick control is an important determinant of co-contraction (Dahl and Altenmüller, 2008; Fujisawa and Miura, 2010), which, in turn, has been connected to varying levels performance accuracy. In a study examining playing strategy and performance experience between amateur and non-drummers, Fujisawa and colleagues found that less skilled drummers played with higher levels of muscular strain (co-contraction) (Fujisawa and Miura, 2010). Furthermore, Kawakami and colleagues demonstrated that the sound and energy of the performance during repeated striking was directly related to the acceleration control of the stick both before and after each hit (Kawakami et al., 2008). Similar variations have been found between striking impulse and tempo control strategies of pianists. The use of such strategies influenced not only the tempo but also the tone of the notes (Furuya and Kinoshita, 2007).

Lastly, in the original article we stated that:

“This may have prevented players (especially those with lower expertise) from fulfilling the task demands resulting in reduced performance accuracy (Dounskaia et al., 1998; Fujii et al., 2011).”

The inclusion of (Dounskaia et al., 1998) does not fall within the context of musical movements.

A correction has been made to section *4.2.2. Performance and Tempo, Paragraph 1*.

Our findings are consistent with previous studies that report performance of expert drummers (measured in CV-ITI) to range between 2 and 5% of an eighth note (Madison, 2000). However, across all drummers, expertise, and exercises we observed a significant drop in performance as tempo increased. A possible explanation for this is that we are reaching the biomechanical limits for rapid upper arm movement. The maximum tapping frequency of motor effectors are reported to be between 5 and 7 Hz, corresponding to ITIs of 150–200 ms. The highest tempo in the present study is 400 HPM, which represents an ITI of 150 ms. This may have prevented players (especially those with lower expertise) from fulfilling the task demands resulting in reduced performance accuracy (Fujii et al., 2011).

The authors apologize for these errors and state that they do not change the scientific conclusions of the article in any way. The original article has been updated.

## Publisher's Note

All claims expressed in this article are solely those of the authors and do not necessarily represent those of their affiliated organizations, or those of the publisher, the editors and the reviewers. Any product that may be evaluated in this article, or claim that may be made by its manufacturer, is not guaranteed or endorsed by the publisher.
